# Fluoroquinolone prophylaxis does not increase risk of neuropathy in children with acute lymphoblastic leukemia

**DOI:** 10.1002/cam4.3249

**Published:** 2020-07-25

**Authors:** Seth E. Karol, Yilun Sun, Li Tang, Ching‐Hon Pui, Jose Ferrolino, Kim J. Allison, Shane J. Cross, William E. Evans, Kristine R. Crews, Sima Jeha, Joshua Wolf

**Affiliations:** ^1^ Department of Oncology St. Jude Children's Research Hospital Memphis TN USA; ^2^ Department of Biostatistics St. Jude Children's Research Hospital Memphis TN USA; ^3^ Department of Pediatrics University of Tennessee Health Science Center Memphis TN USA; ^4^ Department of Infectious Diseases St. Jude Children's Research Hospital Memphis TN USA; ^5^ Department of Pharmaceutical Sciences St. Jude Children's Research Hospital Memphis TN USA

**Keywords:** child, ciprofloxacin, leukemia, lymphoid, levofloxacin, polyneuropathy, prophylaxis, antibiotic

## Abstract

**Background:**

Fluoroquinolone antibiotics are frequently utilized in pediatric oncology patients as prophylaxis or step‐down therapy following broad spectrum beta‐lactam therapy for febrile neutropenia. Concerns regarding neurotoxicity limit the use of these agents. No studies have evaluated the association between fluoroquinolone use and neurotoxicity in pediatric oncology patients receiving other neurotoxic agents such as vincristine.

**Methods:**

An observational cohort study comprising patients aged 0‐18 at diagnosis enrolled on a prospective study for treatment of acute lymphoblastic leukemia (ALL) at a pediatric comprehensive cancer center between October 2007 and November 2018. Data for neuropathic pain and sensory or motor neuropathy were collected prospectively, and a Cox proportional hazards regression model was used to evaluate associations between administration of fluoroquinolone antibiotics during induction therapy and subsequent development of vincristine‐induced peripheral neurotoxicity (VIPN).

**Results:**

A total of 598 participants were enrolled, including 338 (57%) who received fluoroquinolones during induction therapy; of these 470 (79%) were diagnosed with VIPN and 139 (23%) were diagnosed with high‐grade (Grade 3+) VIPN. On unadjusted analyses, and analyses adjusted for age and race, there was no evidence of an association between fluoroquinolone exposure and subsequent VIPN (hazard ratio [HR] 0.8, 95% CI 0.5‐1.04, *P* = .08) or high‐grade VIPN (HR 1.1, 95% CI 0.4‐2.2, *P* = .87).

**Conclusions:**

The results of this observational study do not show an association between exposure to fluoroquinolone antibiotics during induction therapy for ALL and subsequent development of vincristine‐induced peripheral neuropathies, and suggest that a large increase in VIPN is unlikely.

## Graphical abstract

Both vincristine and fluoroquinolone antibiotics can cause peripheral neurotoxicity, but it is not known whether coadministration of fluoroquinolones increases the risk of vincristine‐induced peripheral neurotoxicity (VIPN). In this cohort study of 598 pediatric patients undergoing treatment for acute lymphoblastic leukemia, VIPN was common, but fluoroquinolone prophylaxis during induction therapy did not increase the risk.

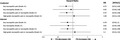

## 1 INTRODUCTION

Fluoroquinolone antibiotics are frequently used in patients with cancer as prophylaxis or step‐down therapy following episodes of febrile neutropenia.^1^, ^2^, ^3^, ^4^ Such prophylaxis has become increasingly common in children with acute lymphoblastic leukemia (ALL) because it may prevent bacterial infections that are otherwise frequent, life‐threatening, and contribute to other long‐term sequelae such as renal insufficiency, neurocognitive dysfunction, and osteonecrosis.^5^, ^6^, ^7^, ^8^, ^9^ Concerns about toxicity contribute to the limited use of fluoroquinolone prophylaxis.

Fluoroquinolone antibiotics are known to be neurotoxic, causing a 1.5‐2‐fold increase in risk of peripheral neuropathy in adults. Whether such neurotoxicity occurs in children receiving other neurotoxic agents is unknown. Vincristine is a vinca‐alkaloid chemotherapy agent and a core component of curative therapy for ALL and many other malignancies.^10^ Vincristine‐induced peripheral neurotoxicity (VIPN) can lead to acute or long‐term neuropathic pain, muscle weakness or sensory deficits, which in turn can cause significant disability.^11^ The mechanism for VIPN is complex and likely related to inhibition of microtubule aggregation, cell membrane remodeling, disruption of calcium homeostasis, and local inflammation.^12^ Risk factors for VIPN include cumulative vincristine dose, older age, Caucasian race, and specific genetic polymorphisms.^13^ It has been long postulated that fluoroquinolones might potentiate VIPN when coadministered with vincristine.^14^ Although there is no known pharmacokinetic interaction, there are several reasons to expect an adverse interaction between fluoroquinolones and VIPN. The US Food and Drug Administration released a 2013 enhanced warning regarding fluoroquinolone‐induced peripheral neuropathy,^15^ and several studies suggest that fluoroquinolones approximately double the risk of peripheral neuropathy in adults.^16^, ^17^, ^18^, ^19^ The effect of fluoroquinolones on VIPN might be additive, leading to unmasking or exacerbation of VIPN, or synergistic due to the direct interaction between the pathological effects of both drugs.^20^, ^21^

The effects of coadministration of other neurotoxic medications on risk of VIPN is poorly understood. To address this important research gap, we undertook a retrospective cohort study to determine the association between fluoroquinolone exposure and subsequent development of neuropathic pain, or sensory and motor neuropathy in children and adolescents receiving vincristine for treatment of ALL.

## 2 PATIENTS AND METHODS

This was an observational cohort study of patients receiving therapy for newly diagnosed B‐cell precursor or T‐ALL in a prospective study at a pediatric comprehensive cancer center in the USA between October 2007 and 1 November 2018. Adverse event data, demographics, and cancer treatment were documented prospectively by the study team, and Common Terminology Criteria for Adverse Events version 3.0 was used to classify and rate the severity of adverse events. Neuropathic pain and other sensory or motor neuropathies were diagnosed clinically. Data describing antibiotic exposure during induction therapy were collected retrospectively from the medical record. The study was approved by the Institutional Review Board.

### 2.1 Inclusion and exclusion criteria

All patients enrolled on the TOTAL16 study for newly diagnosed ALL who were aged from birth to 18 years at diagnosis at St. Jude Children's Research Hospital between 29 October 2007 and 1 April 2017 were eligible for inclusion. Details of this population have been previously published.^22^

### 2.2 Treatment protocol

Induction therapy for leukemia was administered over 42 days and included four weekly doses of vincristine (1.5 mg/m^2^, maximum 2 mg), 4 weeks of prednisone, plus other chemotherapy; a further 29 (low risk) or 30 (standard or high risk) doses of vincristine (1.5‐2 mg/m^2^, maximum 2 mg) were administered over the subsequent 109 weeks. During induction therapy some participants received antibacterial prophylaxis with levofloxacin or ciprofloxacin throughout periods of neutropenia (ANC < 500). Fluoroquinolone prophylaxis was initially prescribed according to clinician preference but was made standard for all enrolled participants from August 2014. All patients were assessed weekly for toxicity throughout all phases of therapy (at least twice weekly during induction and at least weekly thereafter) using treating clinician exam and parental report of symptoms; because a prospective interventional study for management of neuropathic pain (NCT01506453) was activated in September 2011 and may have affected ascertainment of cases of neuropathic pain, treatment era (before or after 1 September 2011) was considered a potential confounder.

### 2.3 Statistical analysis

To evaluate the association between exposure to fluoroquinolone antibiotics during induction therapy and subsequent development of neuropathic pain or other sensory/motor neuropathy (subsequently referred to as neuropathy), we developed a Cox proportional hazards regression model which included fluoroquinolone exposure as a time‐dependent variable and neuropathy as the outcome variable. Cases were censored at the first occurrence of neuropathy and controls were censored at the time of discontinuation of protocol therapy: completion of treatment, death from any cause, relapse, or hematopoietic stem cell transplantation. An unadjusted regression model included only fluoroquinolone exposure, and an adjusted multivariable model accounted for treatment era plus other relevant confounders. We separately considered the association of fluoroquinolones with any grade of symptomatic neuropathy (Grade 2+; interfering with function but not interfering with activities of daily living), any grade of symptomatic neuropathic pain (Grade 2+), any grade of symptomatic neuropathy or neuropathic pain (Grade 2+), and high‐grade neuropathy or neuropathic pain (Grade 3+; interfering with activities of daily living). We also performed analyses restricted to onset of neurotoxicity within the first 90 days of leukemia therapy.

We considered several potential confounders for inclusion in the multivariable regression model; these included age at diagnosis (dichotomized at median), leukemia risk category, self‐reported race, and sex. Each variable was separately evaluated using a univariate Cox proportional hazards regression model, and variables associated with neuropathic pain or neuropathy in univariate analysis were further included in the multivariable model.

Because levofloxacin was used with higher frequency than ciprofloxacin, we aimed to evaluate the potential effect of levofloxacin by comparing the relative effect of ciprofloxacin vs levofloxacin, and no fluoroquinolone vs levofloxacin, on neuropathic pain or neuropathy using Cox proportional hazard regression models as described above. Because almost all participants who received levofloxacin prophylaxis were treated in the later era, this analysis was restricted to patients enrolled after 1 September 2011. All statistical analyses were performed using SAS 9.4 (SAS Institute).

## 3 RESULTS

There were 598 evaluable participants, with a median duration of study follow‐up of 2.6 years. Participant characteristics are shown in Table 1. Of these, 463 (77.4%) completed all protocol chemotherapy, 57 (9.5%) were still receiving chemotherapy at the time of analysis, and 78 (13.0%) discontinued the study early for the reasons shown in Table S1. Neuropathy and neuropathic pain were common. (Table 1). Neuropathy and neuropathic pain were significantly associated with age at diagnosis >6 years and white race, but not with sex, or leukemia risk category. (Table S2) Therefore race (white vs all others) and age (above and below median) were included as covariates in adjusted models.

**TABLE 1:** Characteristics of study participants

Participant characteristics	Fluoroquinolone exposure (N = 338)	No fluoroquinolone exposure (N = 260)	All participant (N = 598)
	n (%)	n (%)	n (%)
Median age, y (IQR)	6.3 (3.5, 10.9)	5.5 (3.0, 9.9)	6.0 (3.2, 10.6)
Age group			
≤ 6 y	160 (47.3)	138 (53.1)	298 (49.8)
> 6 y	178 (52.7)	122 (46.9)	300 (50.2)
Sex			
Female	145 (42.9)	103 (39.6)	248 (41.5)
Male	193 (57.1)	157 (60.4)	350 (58.5)
Self‐declared race			
White	266 (78.7)	198 (76.2)	464 (77.6)
Black	48 (14.2)	40 (15.4)	88 (14.7)
Other	24 (7.1)	22 (8.5)	46 (7.7)
Leukemia subtype			
B cell	277 (82)	217 (83.5)	494 (82.6)
T cell	61 (18)	43 (16.5)	104 (17.4)
Treatment risk group			
Low	147 (43.5)	114 (43.8)	261 (43.6)
Standard	149 (44.1)	129 (49.6)	278 (46.5)
High	42 (12.4)	17 (6.5)	59 (9.9)
Fluoroquinolone exposure during induction			
Any fluoroquinolones	338 (100)	N/A	338 (56.5)
Levofloxacin	192 (56.8)	N/A	192 (32.1)
Ciprofloxacin	163 (48.2)	N/A	163 (27.3)
Neuropathic complication			
Neuropathic pain (Grade 2+)	250 (74)	191 (73.5)	441 (73.7)
Peripheral neuropathy (Grade 2+)	95 (28.1)	72 (27.7)	167 (27.9)
Any neuropathy or neuropathic pain (Grade 2+)	269 (79.6)	201 (77.3)	470 (78.6)
High‐grade neuropathy or neuropathic pain (Grade 3+)	73 (21.6)	66 (25.4)	139 (23.2)
Early neuropathic complication (≤90 d)			
Neuropathic pain (Grade 2+)	128 (37.9)	101 (38.8)	229 (38.3)
Peripheral neuropathy (Grade 2+)	24 (7.1)	20 (7.7)	44 (7.4)
Any neuropathy or neuropathic pain (Grade 2+)	140 (41.4)	108 (41.5)	248 (41.5)
High‐grade neuropathy or neuropathic pain (Grade 3+)	29 (8.6)	18 (6.9)	47 (7.9)

Note

Early neuropathic complication, neuropathic complication identified within 90 d after initiation of therapy for ALL

Abbreviation: ALL, acute lymphoblastic leukemia.

**FIGURE 1:**
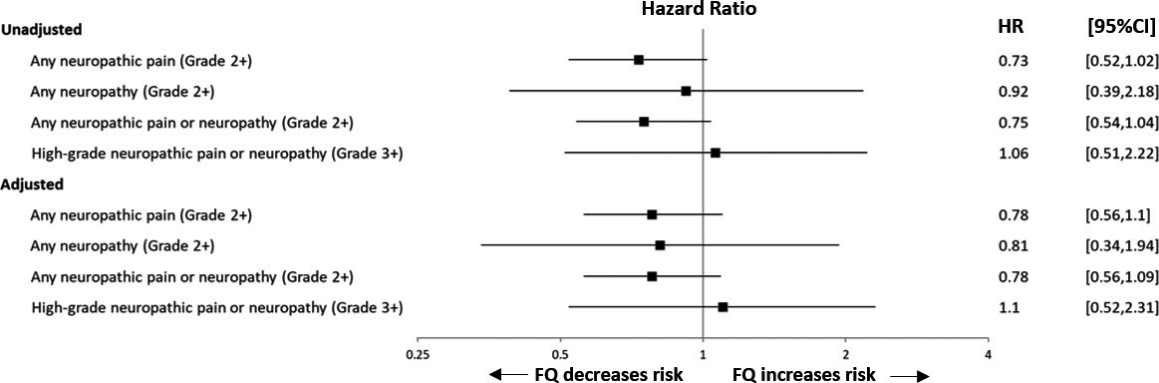
Effect of fluoroquinolone exposure on hazard of neuropathic pain or neuropathy. FQ, fluoroquinolone

Median fluoroquinolone exposure in participants who received levofloxacin or ciprofloxacin was 22 days (IQR 12‐34 days). In unadjusted Cox proportional hazards regression models, exposure to fluoroquinolone antibiotics during induction therapy was not associated with an increase in neuropathic pain, neuropathy, combined pain/neuropathy, or high‐grade neuropathy or neuropathic pain. (*P* = .066, hazard ratio [HR], 95% confidence interval [95% CI] 0.52‐1.02; *P* = .852, HR 0.92, 95% CI 0.39‐2.18; *P* = .084, HR 0.75, 95% CI 0.54‐1.04; *P* = .872, HR 1.06, 95% CI 0.51‐2.22, respectively). In analyses adjusting for race and age, fluoroquinolone exposure in induction was not associated with neuropathic pain, neuropathy, combined pain/neuropathy, or high‐grade neuropathy or neuropathic pain (*P* = .162, HR 0.78, 95% CI 0.56‐1.1; *P* = .64, HR 0.81, 95% CI 0.34‐1.94; *P* = .141, HR 0.78, 95% CI 0.56‐1.09; *P* = .803, HR 1.1, 95% CI 0.52‐2.31 respectively; Figure 1; Table S3; Figure S1). This lack of association was maintained after restriction to early onset symptoms. (*P* = .084, HR 0.75, 95% CI 0.54‐1.04 for unadjusted analysis; *P* = .23, HR 0.81, 95% CI 0.58‐1.14 for adjusted analysis; Figure S2).

Considering levofloxacin alone, there was no significant increase in neuropathy or neuropathic pain when participants receiving levofloxacin were compared to those receiving either ciprofloxacin or no fluoroquinolone antibiotic for any of the outcomes of interest. (Table S4).

## 4 DISCUSSION

Long‐term survival for children and adolescents with ALL now exceeds 90% in high income countries.^23^ However, toxicities associated with anticancer chemotherapy are frequent and important contributors to poor quality‐of‐life in survivors. The most commonly identified acute and persistent toxicity of vincristine is peripheral neurotoxicity.^24^ Identifying features that affect risk of VIPN might help reduce the frequency or severity of this complication. Identification of genetic polymorphisms that predispose to VIPN has led to proposals for genotype‐guided dose‐modification (NCT03117751), and the discovery that azole antifungal drugs increase vincristine exposure has led to avoidance of those drugs around the time of vincristine administration.^25^, ^26^ Similarly, evidence that exposure to fluoroquinolone antibiotics increased the risk of VIPN would aid clinician decision‐making. In this relatively large study, we did not find any evidence that administration of fluoroquinolone antibiotics, especially levofloxacin, during induction therapy for ALL increased the short‐ or long‐term risk of neuropathic pain or sensory/motor peripheral neuropathy. This adds to evidence from the Children's Oncology Group which did not identify an increase in musculoskeletal complications in pediatric oncology patients receiving levofloxacin for antibacterial prophylaxis.^4^

The study has some limitations. Although we attempted to identify and control for potential confounders, it is possible that an unrecognized confounder exists. The diagnosis of neuropathy was based on clinician evaluation and not on more objective approaches, such as nerve conduction studies.^21^ Similarly, alternative explanations for peripheral neuropathy, such as vitamin deficiency, were investigated only at the discretion of the treating clinician, and might have been missed. Finally, because fluoroquinolone prophylaxis decreased the incidence of *Clostridioides difficile* infection,^3^ small increases in neuropathy from fluoroquinolones may have been offset by decreased neuropathy from a reduction in metronidazole use.

The strengths of this study include the relatively large sample size, prospective identification and grading of peripheral neuropathy, direct confirmation of fluoroquinolone prescribing and administration from the medical record, long‐term rigorous clinical follow‐up, high‐resolution information about chemotherapy received, and a careful approach to statistical analysis. The use of Cox regression including fluoroquinolone exposure as a time‐dependent variable allowed us to account for all identified confounders and for peripheral neuropathy that occurred before or after exposure to fluoroquinolones. We also evaluated the potential of a short‐term (up to 90 days) and long‐term (up to 2.5 years) association between fluoroquinolones and peripheral neuropathy in separate analyses to account for a possible delay in identification or onset of the symptoms.

## 5 CONCLUSIONS

In this this study, we found that fluoroquinolone prophylaxis during induction therapy for ALL did not increase the risk of peripheral neurotoxicity in children and adolescents receiving vincristine weekly for 4 weeks and every 4 weeks during continuation. Although this is reassuring, the risks and benefits of fluoroquinolone prophylaxis in this population still require more evaluation. Further studies that include long‐term follow‐up for toxicity and antimicrobial resistance are planned.

## CONFLICTS OF INTEREST

The authors have no conflicts of interest relevant to this article to disclose.

## AUTHOR CONTRIBUTION

Dr Karol conceptualized and designed the study and reviewed and edited the manuscript. Mr Sun and Dr Tang designed and performed the statistical analyses and reviewed and edited the manuscript. Dr Pui, Dr Jeha, Dr Evans, and Dr Crews designed the larger interventional study (TOTXVI) and reviewed and edited the manuscript. Dr Ferrolino and Ms Allison collected the data and reviewed and edited the manuscript. Dr Cross designed the study and reviewed and edited the manuscript. Dr Wolf conceptualized and designed the study, collected the data, designed and performed the analyses, created the figures, and wrote the manuscript. All authors reviewed and approved the final manuscript as submitted and agree to be accountable for all aspects of the work.

## Supporting information

Supplementary Material

## Data Availability

De‐identified data used in the preparation of this manuscript are available upon request.
