# Supplementary material for: Fluoroquinolone prophylaxis does not increase risk of neuropathy in children with acute lymphoblastic leukemia
Source: Cancer Med. 2020 Jul 25;9(18):6550–5. doi: 10.1002/cam4.3249 (PMC7520302; doi:10.1002/cam4.3249)

**Supplemental Table 1.** Reasons for study discontinuation (n = 78)

| Reason for discontinuation | n | (%) |
| --- | --- | --- |
| Toxicity |  |  |
| Liver disease | 4 | (0.7%) |
| Extensive molluscum | 2 | (0.3%) |
| Encephalopathy | 1 | (0.2%) |
| Neuropathy/Myopathy | 1 | (0.2%) |
| Pancreatitis with pseudocyst | 1 | (0.2%) |
| Varicella encephalitis | 1 | (0.2%) |
| Other toxicity | 1 | (0.2%) |
| Injury | 1 | (0.2%) |
| Death | 26 | (4.3%) |
| Other |  | (0%) |
| Transfer for HCT | 21 | (3.5%) |
| Lost to follow-up | 1 | (0.2%) |
| Relapse | 6 | (1.0%) |
| Parent request | 9 | (1.5%) |
| Other reason | 3 | (0.5%) |
| Total | 78 | (13.0%) |

HCT, hematopoietic stem cell transplantation; (%), percentage of all participants

**Supplemental Table 2.** Association between potential confounding variables and neuropathic pain or neuropathy

|  | Any neuropathic pain  (Grade 2+) | | | Any neuropathy  (Grade 2+) | | | Any neuropathic pain or neuropathy  (Grade 2+) | | | High-grade neuropathic pain or neuropathy  (Grade 3+) | | |
| --- | --- | --- | --- | --- | --- | --- | --- | --- | --- | --- | --- | --- |
| Variable | HR | 95% CI | P | HR | 95% CI | P | HR | 95% CI | P | HR | 95% CI | P |
| Age in years |  |  |  |  |  |  |  |  |  |  |  |  |
| >6 vs. ≤6 | 1.39 | (1.15, 1.68) | <.001 | 1.22 | (0.90, 1.65) | 0.207 | 1.30 | (1.09, 1.56) | 0.004 | 1.77 | (1.25, 2.48) | 0.001 |
| Sex |  |  |  |  |  |  |  |  |  |  |  |  |
| Male vs. Female | 0.91 | (0.75, 1.09) | 0.305 | 0.91 | (0.67, 1.24) | 0.545 | 0.90 | (0.75, 1.08) | 0.237 | 0.84 | (0.60, 1.17) | 0.303 |
| Race |  |  |  |  |  |  |  |  |  |  |  |  |
| Black vs. White | 0.52 | (0.39, 0.70) | <.001 | 0.38 | (0.21, 0.68) | 0.001 | 0.49 | (0.37, 0.65) | <.001 | 0.60 | (0.34, 1.04) | 0.070 |
| Other vs. White | 0.69 | (0.47, 1.02) | 0.062 | 0.44 | (0.21, 0.94) | 0.035 | 0.66 | (0.45, 0.96) | 0.032 | 0.59 | (0.27, 1.26) | 0.170 |
| Risk Category |  |  |  |  |  |  |  |  |  |  |  |  |
| Low vs. High | 1.17 | (0.81, 1.67) | 0.401 | 0.93 | (0.50, 1.71) | 0.807 | 1.17 | (0.83, 1.66) | 0.370 | 0.63 | (0.34, 1.17) | 0.144 |
| Standard vs. High | 1.24 | (0.86, 1.77) | 0.246 | 1.04 | (0.57, 1.91) | 0.899 | 1.22 | (0.87, 1.72) | 0.253 | 0.92 | (0.51, 1.66) | 0.786 |

The second category is the reference category

**Supplemental Table 3.** Association between fluoroquinolone exposure during induction therapy and neuropathic pain or neuropathy

|  | Any neuropathic pain  (Grade 2+) | | | Any neuropathy  (Grade 2+) | | | Any neuropathic pain or neuropathy  (Grade 2+) | | | High-grade neuropathic pain or neuropathy  (Grade 3+) | | |
| --- | --- | --- | --- | --- | --- | --- | --- | --- | --- | --- | --- | --- |
|  | HR | 95% CI | P | HR | 95% CI | P | HR | 95% CI | P | HR | 95% CI | P |
| Any onset |  |  |  |  |  |  |  |  |  |  |  |  |
| Unadjusted | 0.73 | (0.52, 1.02) | 0.066 | 0.92 | (0.39, 2.18) | 0.852 | 0.75 | (0.54, 1.04) | 0.084 | 1.06 | (0.51, 2.22) | 0.872 |
| Adjusted | 0.78 | (0.56, 1.10) | 0.162 | 0.81 | (0.34, 1.94) | 0.640 | 0.78 | (0.56, 1.09) | 0.141 | 1.10 | (0.52, 2.31) | 0.803 |
| Early onset |  |  |  |  |  |  |  |  |  |  |  |  |
| Unadjusted | 0.73 | (0.52, 1.02) | 0.066 | 0.92 | (0.39, 2.18) | 0.852 | 0.75 | (0.54, 1.04) | 0.084 | 1.06 | (0.51, 2.22) | 0.874 |
| Adjusted | 0.81 | (0.57, 1.15) | 0.239 | 0.91 | (0.38, 2.18) | 0.831 | 0.81 | (0.58, 1.14) | 0.230 | 1.13 | (0.53, 2.39) | 0.760 |

**Supplemental Table 4.** Association between levofloxacin exposure and neuropathic pain or neuropathy

|  | Any neuropathic pain  (Grade 2+) | | | Any neuropathy  (Grade 2+) | | | Any neuropathic pain or neuropathy  (Grade 2+) | | | High-grade neuropathic pain or neuropathy  (Grade 3+) | | |
| --- | --- | --- | --- | --- | --- | --- | --- | --- | --- | --- | --- | --- |
|  | HR | 95% CI | P | HR | 95% CI | P | HR | 95% CI | P | HR | 95% CI | P |
| Ciprofloxacin vs. Levofloxacin | | | | | | | | | | | | |
| Any onset |  |  |  |  |  |  |  |  |  |  |  |  |
| Unadjusted | 0.65 | (0.19, 2.19) | 0.49 | 1.75 | (0.19, 16.10) | 0.621 | 0.61 | (0.18, 2.04) | 0.423 | 1.28 | (0.15, 11.12) | 0.823 |
| Adjusted | 0.57 | (0.17, 1.98) | 0.38 | 1.65 | (0.18, 15.16) | 0.656 | 0.55 | (0.16, 1.87) | 0.334 | 1.09 | (0.13, 9.34) | 0.94 |
| Early onset |  |  |  |  |  |  |  |  |  |  |  |  |
| Unadjusted | 0.65 | (0.19, 2.19) | 0.49 | 1.75 | (0.19, 16.12) | 0.62 | 0.61 | (0.18, 2.04) | 0.423 | 1.28 | (0.15, 11.13) | 0.822 |
| Adjusted | 0.61 | (0.18, 2.08) | 0.426 | 1.64 | (0.18, 14.89) | 0.658 | 0.56 | (0.16, 1.92) | 0.359 | 1.13 | (0.13, 9.71) | 0.909 |
| No fluoroquinolone vs. levofloxaxin | | | | | | | | | | | | |
| Any onset |  |  |  |  |  |  |  |  |  |  |  |  |
| Unadjusted | 1.27 | (0.84, 1.92) | 0.257 | 1.72 | (0.48, 6.14) | 0.402 | 1.26 | (0.84, 1.88) | 0.267 | 1.73 | (0.57, 5.30) | 0.335 |
| Adjusted | 1.28 | (0.84, 1.94) | 0.251 | 1.74 | (0.49, 6.21) | 0.39 | 1.27 | (0.85, 1.90) | 0.251 | 1.66 | (0.54, 5.09) | 0.377 |
| Early onset |  |  |  |  |  |  |  |  |  |  |  |  |
| Unadjusted | 1.27 | (0.84, 1.92) | 0.257 | 1.72 | (0.48, 6.15) | 0.401 | 1.26 | (0.84, 1.88) | 0.267 | 1.73 | (0.57, 5.31) | 0.334 |
| Adjusted | 1.29 | (0.85, 1.95) | 0.232 | 1.75 | (0.49, 6.21) | 0.388 | 1.27 | (0.85, 1.90) | 0.24 | 1.65 | (0.53, 5.15) | 0.387 |

**Supplemental figures**

**Supplemental Figure 1.** Cumulative incidence of neuropathic pain or neuropathy in participants according to induction fluoroquinolone exposure


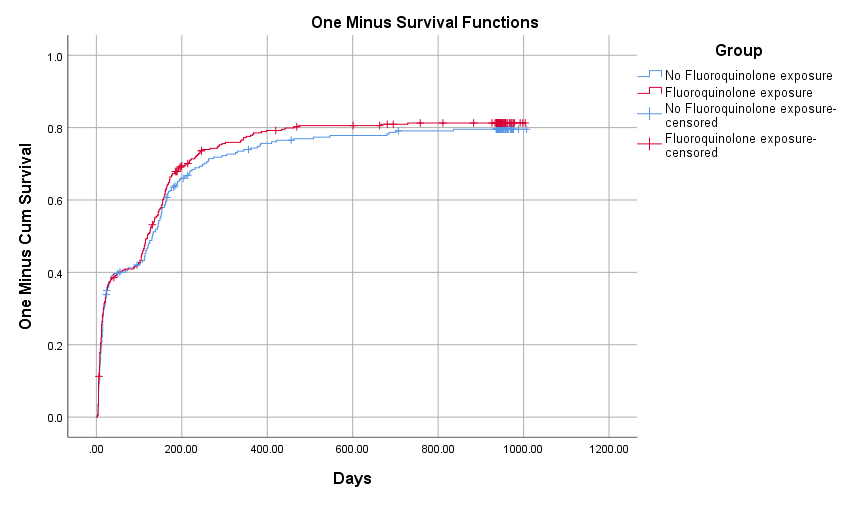

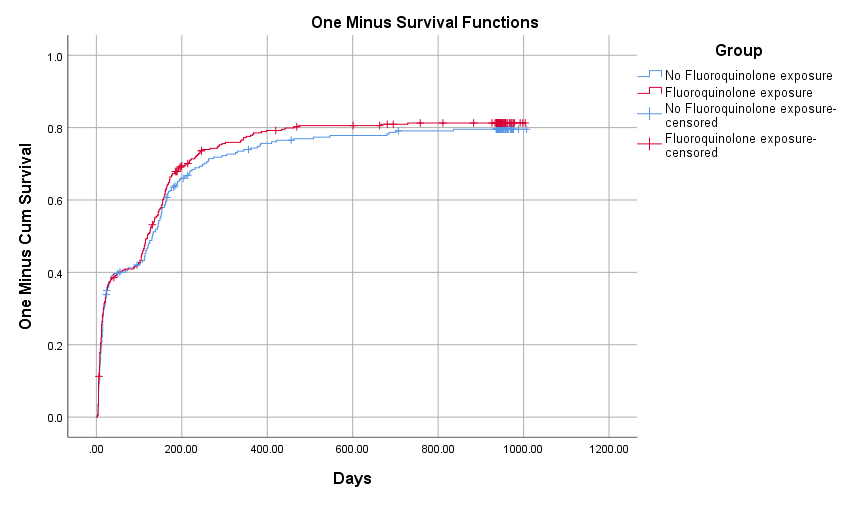


**Cumulative incidence of neuropathy or neuropathic pain**

Proportion affected

Time from diagnosis (days)

**Supplemental Figure 2.** Effect of fluoroquinolone exposure on hazard of *early onset* neuropathic pain or neuropathy


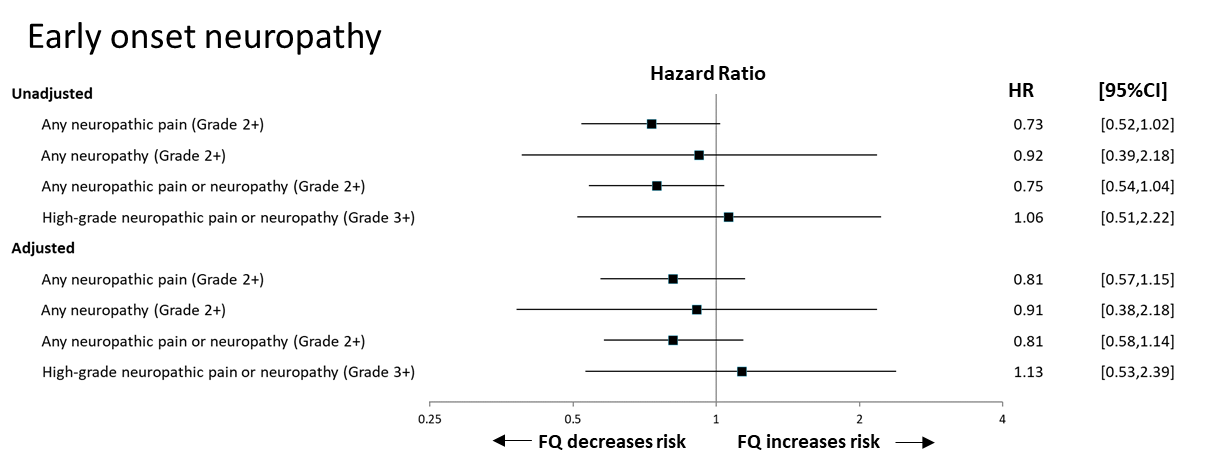

Supplement: Supplementary file 1 — Supplementary Material [file CAM4-9-6550-s001.docx]
